# Stochastic flux analysis of chemical reaction networks

**DOI:** 10.1186/1752-0509-7-133

**Published:** 2013-12-07

**Authors:** Ozan Kahramanoğulları, James F Lynch

**Affiliations:** 1 The Microsoft Research - University of Trento, Centre for Computational and Systems Biology, Trento, Italy; 2 Department of Computer Science, Clarkson University, Potsdam, NY, USA

**Keywords:** Chemical reaction networks, Flux, Stochastic simulation, Markov Chains, Rho GTP-binding proteins, Oyster reef, Oscillator, Phosphorelay

## Abstract

**Background:**

Chemical reaction networks provide an abstraction scheme for a broad range of models in biology and ecology. The two common means for simulating these networks are the deterministic and the stochastic approaches. The traditional deterministic approach, based on differential equations, enjoys a rich set of analysis techniques, including a treatment of reaction fluxes. However, the discrete stochastic simulations, which provide advantages in some cases, lack a quantitative treatment of network fluxes.

**Results:**

We describe a method for flux analysis of chemical reaction networks, where flux is given by the flow of species between reactions in stochastic simulations of the network. Extending discrete event simulation algorithms, our method constructs several data structures, and thereby reveals a variety of statistics about resource creation and consumption during the simulation. We use these structures to quantify the causal interdependence and relative importance of the reactions at arbitrary time intervals with respect to the network fluxes. This allows us to construct reduced networks that have the same flux-behavior, and compare these networks, also with respect to their time series. We demonstrate our approach on an extended example based on a published ODE model of the same network, that is, Rho GTP-binding proteins, and on other models from biology and ecology.

**Conclusions:**

We provide a fully stochastic treatment of flux analysis. As in deterministic analysis, our method delivers the network behavior in terms of species transformations. Moreover, our stochastic analysis can be applied, not only at steady state, but at arbitrary time intervals, and used to identify the flow of specific species between specific reactions. Our cases study of Rho GTP-binding proteins reveals the role played by the cyclic reverse fluxes in tuning the behavior of this network.

## Background

Chemical reaction networks are broadly used as a representation scheme for modeling and simulating a variety of systems from biochemical interactions at the molecular level to higher-level mechanisms. In ecology, some individual-based models enjoy compact representations in the form of chemical reaction networks. Examples of these include Lotka-Volterra predator-prey systems [1,2]^a^ and plant-pollinator systems [3]

Simulations on chemical reaction networks can be performed by resorting to various techniques and tools that implement deterministic or stochastic approaches. Deterministic or stochastic approaches thus provide the two main ways of modeling systems governed by mass-action kinetics, and offer different advantages. The more traditional deterministic method uses ordinary differential equations to approximate the changes in population sizes. While this approach benefits from a greater availability of analysis techniques, it ignores the fundamental discrete and stochastic nature of the reactions, and this can be important, especially for smaller population sizes that are frequently seen in biological systems. In this respect, stochastic simulations provide advantages, for example, in capturing the intrinsic noise in the biochemical systems [4], or species extinctions in the ecosystem models [5].

With respect to the stochastic approach, chemical reaction networks are commonly mapped to a language with a stochastic simulation capability. For example, various implementations of stochastic Petri nets (see, e.g., [6]), which are isomorphic to chemical reaction networks, provide a straight-forward means for this. However, the analysis techniques on stochastic simulations of reaction networks are still underdeveloped in comparison to the rich arsenal of differential equation analysis techniques that have their roots in Newton’s physics. In particular, flux analysis on chemical reaction networks with differential equation representations are well established. Within the deterministic setting, there is a growing number of studies on flux analysis that include issues related to simplification of models [7], while a stochastic treatment of flux is still lacking.

In this paper, we study a class of Markov chains that typically emerge in the stochastic simulations of chemical reaction networks. In these dynamical networks, the states are populations of agents of various species, and the state transitions are updates to subpopulations of the state. For example, in a model of classical chemical kinetics, there are finitely many chemical species, and the states are finite multisets of species. Transitions are described by a finite set of reactions of the form

(1) $$m_{1}R_{1}+\cdots +m_{l}R_{l}\overset{\rho }{\to }n_{1}P_{1}+\cdots +n_{r}P_{r}$$

where the reactants are *R*_1_,…,*R*_*l*_, the products are *P*_1_,…,*P*_*r*_, each *m*_*i*_ is the number of instances of reactant *R*_*i*_ consumed by the reaction, and each *n*_*i*_ is the number of instances of product *P*_*i*_ produced by the reaction. For a particular choice of *m*_1_ reactants of species *R*_1_, *m*_2_ reactants of type *R*_2_, and so on, the probability that they react according to (1) in an infinitesimal time interval *dt* is *ρdt*. The *ρ* is sometimes called the stochastic rate constant.

Mass-action kinetics is based on the assumption that the likelihood of reaction (1) occurring during a small time interval *dt* is *ρdt* multiplied by *k*, the number of ways of choosing the reactants [8,9]. The term *ρk* is often called the total reaction rate or, in the chemical physics literature, the propensity.

We present a method for flux analysis in stochastic simulations with reaction networks, where flux is the flow of resources between reactions of the network. Each simulation is a trajectory of a Markov chain, which is a sequence of computations of the underlying transition system. The trajectory imposes a total order on the transitions of the simulation trajectory that is emphasized by the unique time stamps of the individual transition instances. In this respect, a simulation on a model can be seen as reduction of a complex structure, that is, the model, into a simpler structure, that is, the simulation trajectory. However, during this reduction some of the information on the model is lost, and some is made implicit.

The idea here is to recover this implicit information: when these transitions are inspected from the point of view of their dependencies on one another, it is possible to relax the total order of the transitions into a partial order structure. We can then use this partial order, which we call simulation trace, as a representation of causal dependencies in the simulation, and process the simulation trace to observe the flux in the network with respect to the flow of the resources during simulation from a reaction to another.

Based on these ideas, our method constructs several data structures from the log of the simulations with models that are designed to disclose otherwise implicit resource flow information. These structures hence reveal a variety of statistics about resource creation and consumption during the simulation. We use these structures to quantify the causal interdependence and relative importance of the reaction instances. This allows us to compare simulations at arbitrary time intervals, and use this information, for example, to construct reduced models that have the same behavior with respect to the flow of resources.

We validate our approach with an extended example that is based on a published ordinary differential equations model of the Rho GTP-binding proteins [10], and its stochastic version with its time series analysis given in [11]. Using a deterministic analysis, the model in [10] provides an explanation of the experimentally observed rapid cycling of the Rho GTP-binding proteins between their GDP-bound off states and GTP-bound on states while displaying high activity with respect to the relative concentration of the active GTP-bound Rho proteins. Our stochastic flux analysis confirms the observations of the deterministic analysis of [10] and extends the analysis of [11] with network fluxes. Moreover, it also provides observations that complement those provided by [10]. This is because our stochastic flux analysis makes it possible to quantify the flow of specific species between specific reactions at arbitrary time intervals. As this capability delivers a quantification of the specific resource distributions after being produced by reactions, it exposes the contribution of specific resource flows to system dynamics. For instance, the effect of reverse reactions that can produce counteracting reaction fluxes in a context of multiple reactions can be better observed. This makes it possible to single out the cases with respect to different initial conditions, in which the fluxes shift the simulation resources, and thereby tune the behavior of the network.

In the following, we first introduce our notion of flux on chemical reaction networks, and illustrate the definitions on a simple example network. We then illustrate these concepts on larger networks, which clearly show the distinguishing features of our approach at work. Besides the Rho GTP-binding proteins network, we apply our approach on an oscillator model [12], the Oyster Reef ecosystem model [13,14], and a phosphorelay model [15]. The contribution of our method to the analysis of chemical reaction networks and the example models below that illustrate these concepts can thus be summarized as follows: (*i*.) The notion of flux defined here applies to discrete, stochastic models, and differs from the conventional definition of flux, which is applied instead to continuous, deterministic models, e.g., Rho GTP-binding proteins model. This provides an advantage also in modeling biological systems with smaller population sizes, for which discrete, stochastic models are often more realistic than continuous, deterministic models, e.g., the Oyster Reef model. (*ii*.) The stochastic flux applies not only to steady state, but to arbitrary time intervals of the systems, which are not required to be in a steady state, e.g., the oscillator model and the phosphorelay model. (*iii*.) The stochastic flux shows the amount of each type of resource flowing from any specific reaction to any other reaction, thereby providing an explicit account of causality that cannot be revealed by counting reaction instances, e.g., the Oyster Reef model. In contrast, the conventional version of flux shows only the total amount of all resources generated by each reaction, however it does not distinguish the division of the resources among the reactions that use the resources.

As we show below, the algorithms that implement our stochastic flux analysis are linear in time and space, that is, the time and space requirements of the algorithms are linear functions of the simulation time. The algorithms can thus be included in any discrete event simulator of chemical reaction networks. For the implementation of the models, we used the **SPiM** language and simulation engine [11,16-18], which implements the stochastic *π* calculus. For the flux analysis, we used our tool, written in OCaml, that implements the definitions below.

## Results and discussion

Our method for stochastic flux analysis of chemical reaction networks can be applied to any discrete or continuous time discrete event simulation that implements reaction networks as Markov chains. In the following, we illustrate our method on example networks of models from biology and ecology. For the formal definitions we refer to the Methods section, where the algorithm is described in detail. Below, we first introduce our method on a simple example simulation trajectory with a continuous time Markov chain semantics. As in our case, Gillespie algorithm [19] is commonly used to generate such trajectories of chemical reaction kinetics that implement the law of mass action.

The algorithm is based on marking individuals that are transformed by the reactions, and using the markings to track the causal dependencies between reaction instances during the simulations. We process this causality information to obtain a quantification of the flow of resources between reactions, and thereby providing the network fluxes at chosen time intervals. This is easily implemented by assigning a unique identifier to each network species in the initial state and to each reaction product of every reaction instance. In our case, these identifiers are integers. A reaction instance is a random event whose probability is determined by the current state of the network. A reaction can be applied at a state to obtain a reaction instance if its reactants are available at that state and the reaction is picked by the simulation algorithm from all the applicable reactions. Whenever a reaction is applied at a state the simulation algorithm updates the resulting state with the reaction products and their unique identifiers in a structure that we call simulation trajectory. Because this information can be recorded in a bounded amount of time during simulation in real time, the method does not introduce any additional complexity to the simulation algorithm.

### **Example1**.

Consider the chemical reaction network below, where each reaction is named with an integer.

$$\begin{array}{cc} 1:A\to P+P, & \;2:P\to B,\\ 3:P\to C, & \;4:B+C\to D \end{array}$$

**Figure 1:**
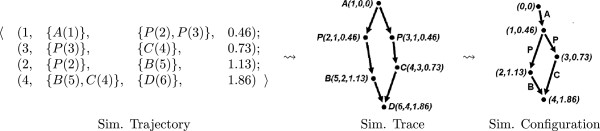
**The transformation from a simulation trajectory generated by the network in Example 1 to its simulation trace, and the transformation from the simulation trace to the simulation configuration.** We first apply Definition 9 and then Definition 10.

The initial state is {*A*(1)}, where 1 is the unique identifier of the species *A*. A possible 4-step simulation trajectory is the sequence of quadruples on the left-hand-side of Figure 1, where the first parameter of the quadruple is the name of the reaction, and the second and third parameters are the sets of the reactants and the products of the reaction instance. The forth parameter is the reaction instance time in the simulation.

By using the unique identifiers of the species in a reaction trajectory, which implicitly indicate the production-consumption relationship between reaction instances of the simulation, we construct a directed graph structure. This graph structure, which we call the simulation trace, makes the causality relationship explicit. In this graph, each node is a species, parameterized with a triple that contains its identifier, the name of the reaction that created it, and the time it is created in the simulation. As an example for a simulation trace, consider the structure in Figure 1, which is obtained from the simulation trajectory of Example 1.

By further processing this graph, we obtain an edge-labeled directed multi-graph that reveals the independence and causality information of the transitions with respect to the flow of specific resources between reactions. The information displayed by this graph is different from the one given by the simulation trace, where the evolution of the species with respect to the reactions are shown. In this graph, which we call simulation configuration, each node is a pair that contains the reaction that is applied and its time in the simulation. Each edge is labeled with the species that is produced by the reaction at the tail of that edge and consumed by the reaction at its head. As an example for this with respect to the simulation trajectory of Example 1, consider the structure on the right-hand-side of Figure 1.

Below, we work with simulation configurations, and process them to obtain flux configurations as formally defined in the Methods section. In the definitions in the Methods section, in order to describe the edges of an edge-labeled graph algebraically, we use 〈*u*,*v*,*l*〉 to denote the edge from vertex *u* to vertex *v* whose label is *l*. Thus in a simulation configuration, the edges are of the form 〈(*j*,*τ*),(*j*^′^,*τ*^′^),*s* 〉, where reaction *j* occurs at time *τ*, and it produces an instance of species *s*, which is consumed by reaction *j*^′^ at time *τ*^′^. Since a reaction may produce several instances of species, the simulation configuration is in general a multi-graph.

Flux configurations are obtained by compressing simulation configurations in order to quantify the flow of resources between the reactions within given time intervals of the simulation. A flux configuration is a graph, where the vertices are the reactions of the network. The edges connect some of these vertices, and each edge has two labels. The first label is a network species, and the second label is a positive integer. A label with a species *s* and number *k* from a reaction *j* to another reaction *j*^′^ means that there are *k* instances of the reaction *j* that deliver the species *s* to reaction *j*^′^. As described in Definition 12, we obtain a flux configuration first by merging the vertices of the simulation configuration such that all the vertices with a certain reaction within the given time interval are mapped to a single vertice by filtering out their time stamps. For each label that denotes a network species, we then count in the simulation configuration the number of edges from each vertice (which corresponds to a reaction of the network) to other vertices within the given time interval. The number of such edges are then used to decorate the edge for that species between the respective reactions.

### **Example2**.

**Figure 2:**
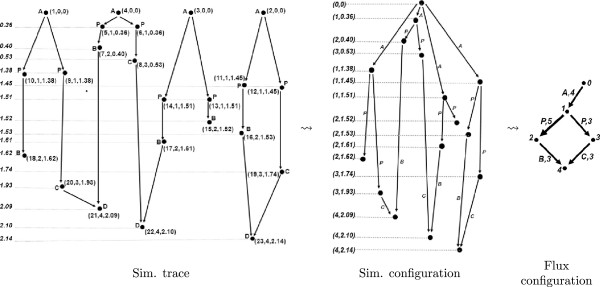
**The simulation trace of a simulation with the network in Example 1.** The initial state is {*A*(1),*A*(2),*A*(3),*A*(4)}. In the simulation trace, each vertex is additionally decorated with its species for illustration purposes. Here, we first apply Definition 10 to obtain the simulation configuration and then Definition 12 to obtain the flux configuration for this trace.

Consider the chemical reaction network given in Example 1. A simulation trace for the initial state {*A*(1),*A*(2),*A*(3),*A*(4)} is depicted on the left-hand-side of Figure 2. The figure demonstrates the simulation configuration and the flux configuration obtained from this trace. In the simulation trace, the vertices are decorated with triples that are respectively the integer identifier of the vertice, identifier of the reaction that created the vertice, and the time of creation. The simulation trace is first mapped to its simulation configuration, where each vertice is a reaction instance and it is denoted with a pair: the first item in the pair is the identifier of the reaction and the second item is the time of creation. The simulation configuration is then mapped to the flux configuration, where the vertices are reactions, and the edges are the pairs of species names and their counts.

A flux configuration provides the information on the intensity of the flow of resources between reactions of the chemical reaction network at given time intervals. This is different from the number of times each reaction fires, because a reaction can receive its resources from different reactions. This also contrasts with the view of flux based on flows between species as they are typically considered in the differential equations setting, which we discuss below.

The time and space complexity of generating the above data structures is linear in the number of simulation steps. This follows from the facts that there is a fixed number of reactions, and each reaction involves a fixed number of species. Consequently, the simulation trace (Definition 8) is a graph of bounded degree, and the number of edges is linear in the number of nodes. Adding each node and its incident edges requires only a bounded number of operations. The simulation configuration (Definition 10) can be constructed in linear time since the projection operators that we use need only access each node and edge of the simulation trace once. It is also evident that the flux graphs can be generated in linear time and space. In fact, the simulation trace can be generated in real time since it adds only bounded time to each simulation step. Because the steps of this algorithm does not modify the generation of the individual events, it can be included in any discrete events simulator of chemical reaction networks.

### A case study: Rho GTP-binding proteins

Rho GTP-binding proteins [10,11,20] serve as molecular switches [21]. Their role can be perceived as regulating the transmission of an incoming signal further to effectors in a molecular module by cycling between inactive and active states, depending on being GDP or GTP bound, respectively. GDP/GTP cycling is regulated by guanine nucleotide exchange factors (GEFs) that promote the GDP dissociation and GTP binding, whereas GTPase-activating proteins (GAPs) have the opposite effect and stimulate the hydrolysis of Rho-GTP into Rho-GDP. In the active GTP-bound state, Rho proteins interact with and activate downstream effectors.

**Figure 3:**
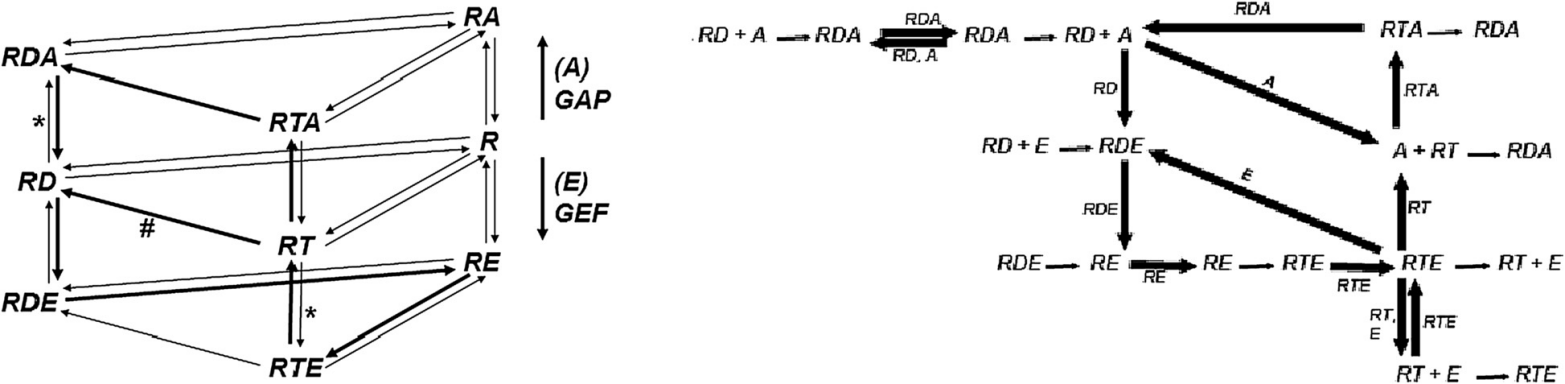
**The structure of the Rho GTP-binding proteins network given in [**[10]**] and the dominant fluxes obtained by stochastic flux analysis on this network. Left:** The arrows denote the reactions of the network. **R** denotes the Rho GTP-binding protein, whereas **RD** and **RT** denote its GDP and GTP bound forms. **A** and **E** denote GAP and GEF. Thus, **RDE**, for example, denotes the protein complex formed by **RD** and **E**. The thick arrows denote the dominant fluxes obtained by the analysis in [10]. **Right:** The dominant fluxes obtained by stochastic flux analysis include the fluxes marked with ∗ and excludes the ones marked with *#* on the left. This analysis indicates also the fluxes due to the enzymes **A** and **E**.

In [10], Goryachev and Pokhilko give an ordinary differential equations (ODE) model of the Rho GTP-binding proteins. The structure of the chemical reaction network of this ODE model is depicted on the left-hand-side of Figure 3: the three forms of the Rho protein (GDP-bound **RD**, GTP-bound **RT**, and nucleotide free **R**) in the middle layer form complexes with GEF (**E**) in the bottom layer and with GAP (**A**) in the top layer. All the reactions except GTP hydrolysis (RT→RD, RTA→RDA, RTE→RDE) are reversible.

With their model Goryachev and Pokhilko provide an explanation of the experimentally observed rapid cycling of the Rho GTP-binding proteins between their GDP-bound off states and GTP-bound on states while displaying high activity, measured by the relative concentration of the GTP-bound Rho proteins (**RT** in Figure 3). In [10], the fluxes are defined for individual reactions with respect to species concentrations and reaction rates such that the reaction flux *J*_*lm*_ that connects species *l* and *m* is defined with respect to the species concentrations and the reaction rate constants. For example, flux *J*_**RD**.**RDE**_ connecting **RD** and **RDE** is *J*_**RD**.**RDE**_ = *k*_**RD**.**RDE**_.**RD**.**E**-*k*_**RDE**.**RD**_.**RDE** where *k*_**RD**.**RDE**_ and *k*_**RDE**.**RD**_ are the corresponding reaction rates.

Goryachev and Pokhilko argue that at large **E**_0_ and **A**_0_ concentrations, only a subset of the reaction fluxes of the network is significant, while the remaining reaction fluxes have negligible values. To test this hypothesis, they introduce a reduced network and provide a comparison with the original network with respect to the flux vectors that substantiate the claim. Goryachev and Pokhilko argue that in the efficient regime the operation of the GEF-GAP control module is given with the cycling loop formed by the union of two linear reaction flux pathways. Given that ⇒ denotes the reaction flux between the species, these two pathways are given as the GEF arm **RD**⇒**RDE**⇒**RE**⇒**RTE**⇒**RT**⇒**RD** and the GAP arm **RT**⇒**RTA**⇒**RDA**⇒**RD**. These pathways are indicated by solid arrows on the left-hand-side of Figure 3. The right-hand-side of Figure 3 puts in comparison the fluxes obtained by stochastic flux analysis as discussed below.

**Figure 4:**
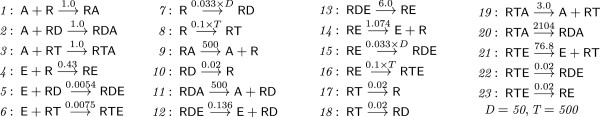
**The GTPase chemical reaction network and their rates as in [**[10]**] and [**[11]**].**

In [11], Cardelli *et al* give a stochastic *π* calculus model of the Rho GTP-binding proteins, which is based on the ordinary differential equations model of [10]. The model in [11], displays an excellent agreement with the ODE model of [10] with respect to the **RT** activity on simulations with varying network structures and different regimes of initial concentrations. In the following, we consider this model for flux analysis. The conversion from the continuous to the stochastic model is explained in [11], where a stochastic model species encodes 1 *μM* of network molecules. With respect to this conversion, in our network we use the same rate values that are used in [10] and [11], listed in Figure 4.

**Figure 5:**
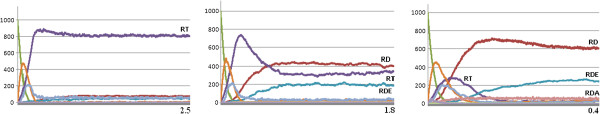
**Example simulation plots of the network in Figure**3**.** The initial numbers of the species are **R**_0_ = 1000 and **E**_0_ = 776. From left to right, the **A**_0_ value is set as 1, 10 and 100. An increase in **A**_0_ in the simulations results in a decrease in the **RT** activity while reducing the recovery time.

The time series analysis indicates that this network is insensitive to the initial **R** levels in terms of activity, given by the **RT**/**R**_0_ ratio at steady state. The network has an activity maximum with the initial concentrations **R** = 1.0 *mM*, **E** = 0.776 *mM* and **A** = 0.66 *μM*[10,11]. In order to analyze the flux of the network at the high activity regime, we ran simulations with **R**_0_ = 1000, **E**_0_ = 776 and **A**_0_ = 1, where we took the closest positive integer number value for **A**_0_ so that factoring of the other simulation parameters would not be required. This results in a near-maximum activity of approx. 0.8 at the steady state with fluctuations due to stochastic simulation. A representative simulation plot with these parameters is in Figure 5 (Left).

**Figure 6:**
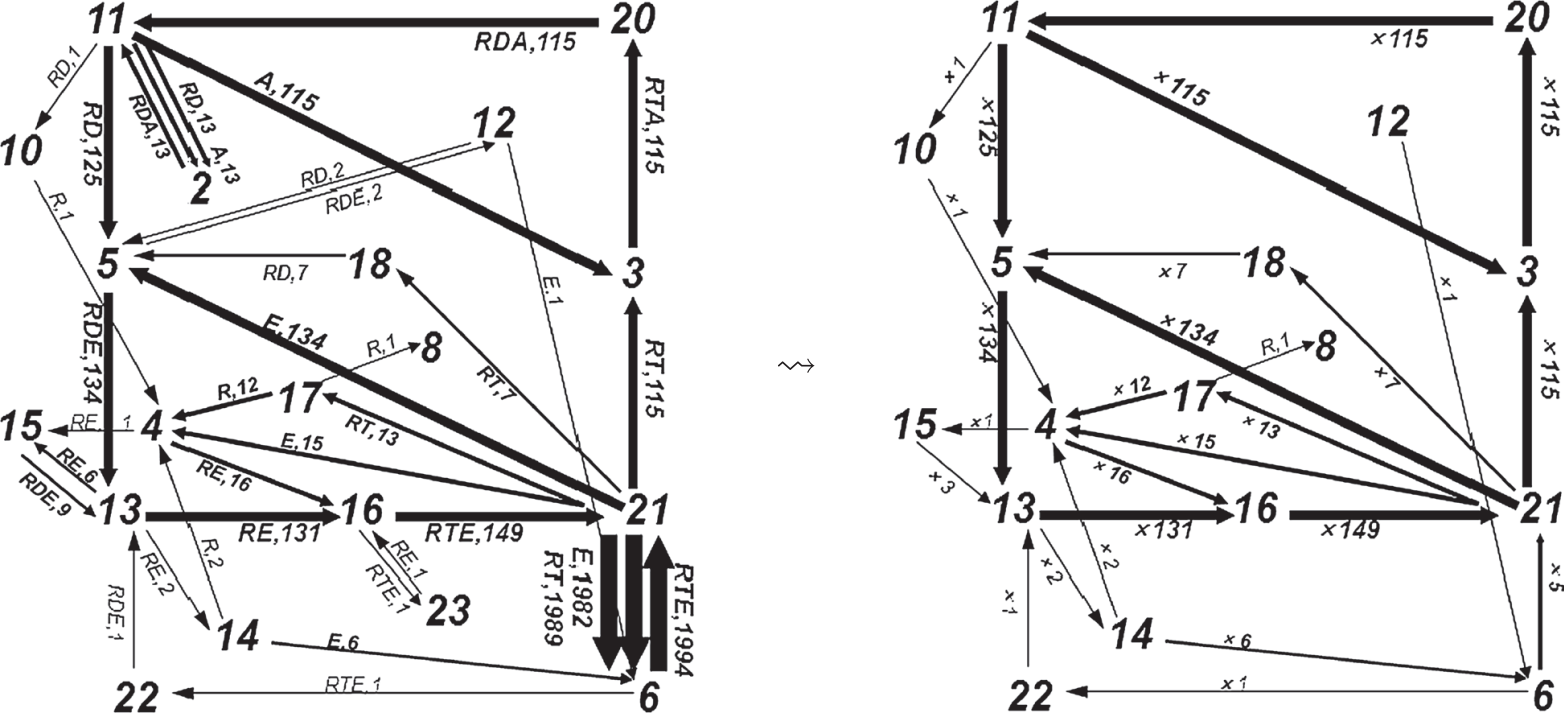
**A representative graphical representations of the structures**F[2,2.5]** and**N[2,2.5]**.** The graphs are obtained from Sim. 3 in Table 1 with respect to the reactions listed in Figure 4.

We analyzed the steady state behavior of the network with respect to the simulations at this regime. For this purpose, we computed the flux for the time interval 2.0<*t*<2.5, that is, F[2,2.5]. This provides a sufficient number of events with respect to the convergence time of the simulation. As with time-series analysis, flux analysis in stochastic simulations needs to be repeated on multiple simulations in order to increase the confidence levels. While some systems require a greater number of simulations, others converge quickly to their steady state as it is the case for the Rho GTP-binding proteins network here. Nevertheless, due to the observations being made on stochastic simulations, we have repeated our analysis on a set of 25 simulations to verify our results, where we repeated the observations discussed below. A representative flux configuration with this network is depicted on the left-hand-side of Figure 6.

**Table 1:** **Simulation results with respect to the average flux of**N[2,2.5]** and the number of fluxes given with number of edges in**N[2,2.5](x)** with respect to various cut-off values**

**Sim.**	**Avg.**	**Cut-off value**x→|N[2,2.5](x)|								
		0.05	0.1	0.15	0.2	0.25	0.3	0.35	0.4	0.45
1	45	17	15	15	13	10	9	9	9	9
2	49	18	16	11	10	10	9	9	9	9
3	40	18	17	15	13	13	12	11	9	9
4	52	18	16	16	12	12	10	10	9	9
5	40	17	17	15	12	9	9	9	9	9
6	52	20	14	11	9	9	9	9	9	9
7	47	17	15	12	11	9	9	9	9	9
8	40	21	16	15	13	11	10	9	9	9
9	45	21	18	15	12	9	9	9	9	9
10	59	17	11	11	10	10	9	9	9	9

The initial number of species are R_0_= 1000, E_0_= 776, A_0_= 1.

In order to compare our flux analysis with the differential equation analysis of [10], we further process the flux configurations to remove the effect of the reverse reactions as this is the case in [10]. These reactions, which we call *cyclic reverse reactions*, are those consisting of a reaction and its reverse, where the products of one are consumed by the other in cycles without having a net flux product for the other reactions in their context. Because the fluxes in [10] are computed by factoring the counter effect of cyclic reverse reactions on reaction fluxes, below we work with net-flux configurations, formally defined in Definition 14 in the Methods section. Net-flux configurations counteract the effect of the cyclic reverse reactions in the flux configurations by taking their difference and mapping them into weighted dags. For the case of reverse reactions that share multiple reactants and products, we consider the maximum flux that is shared by these reactions. The net-flux configuration obtained from a flux-configuration is depicted on the right-hand-side of Figure 6.

In Definition 14, in order to monitor the net influence of each reaction to others, we first obtain a dag  from : whenever there are multiple edges from a reaction to another in , we include in  the edge with the greatest weight by discarding others. We then obtain the dag  from  by taking the difference of the symmetric edges: whenever there is an edge from a reaction *j* to *j*^′^ with weight *m* and an edge from *j*^′^ to *j* with weight *n* such that m is greater than n, we exclude these two edges, and include instead an edge from reaction *j* to *j*^′^ with weight *m*-*n*.

A net-flux configuration provides a summary of the net influence of reactions on each other by counteracting the effect of reverse reactions in the flux configuration and taking the maximum of fluxes, when there are multiple fluxes between two reactions. Although this reduction can reveal further aspects of a network, there are cases where the information removed can denote an important component of the network, as we discuss in the next section.

As a second step for the comparison of our stochastic flux analysis with the deterministic analysis in [10], we reduce the net-flux configurations to dominant fluxes that account for most of the dynamical behavior. For this purpose, we determine a cut-off value that is given by the average of the fluxes as defined below.

#### **Definition3** (average flux).

Given a flux configuration $F[\;t,t^{\prime }]$ with edges $\langle j_{1},j_{1}^{\prime },s_{1},n_{1}\rangle ,\ldots ,\langle j_{\ell },j_{\ell }^{\prime },s_{\ell },n_{\ell }\rangle$, the *average flux* is $\left(\;\overset{\ell }{\underset{k=1}{\sum }}n_{k}\;\right)/\ell \;$. For a net-flux configuration $N[\;t,t^{\prime }]$, the average net-flux is defined analogously.

#### **Definition4** (cut-off).

Given a flux configuration $F[\;t,t^{\prime }]$ and its average flux *σ*, for an $x\in {\mathbb{R}}^{+}$, the *flux after cut-off at x*, denoted by $F[\;t,t^{\prime }](x)$, is the restriction of $F[\;t,t^{\prime }]$ to those edges 〈*j*,*j*^′^,*s*,*n*〉 satisfying *n*>*xσ*. For a net-flux configuration $N[\;t,t^{\prime }]$, we define the net-flux after cut-off at *x*, that is $N[\;t,t^{\prime }](x)$, analogously.

We computed the net flux, N[2,2.5], for the simulations with this network, and applied various cut-off values to compute the net flux after cut off, that is, N[2,2.5](x), for these cut-off values (*x*). Table 1 demonstrates 10 representative simulation results with respect to the average flux and the size of the graph N[2,2.5](x) with respect to various cut-off values for *x*. The size of the graph is here given with the number of its edges. As the cut-off values increase, the size of N[2,2.5](x) converges to 9 fluxes. In Figure 6, we depict F[2,2.5] and N[2,2.5] of Sim. 3 in Table 1. We have chosen Sim. 3 because the cut-off value of this simulation, which results in convergence, is highest among these 10 simulations, and we observe the same behavior as in the other simulations.

**Figure 7:**
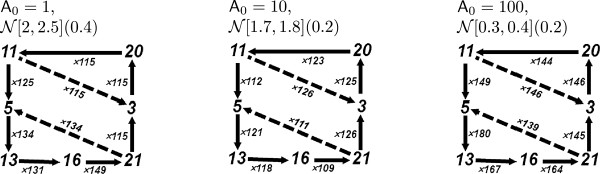
**The graphs displaying the dominant fluxes with respect to the net-flux configurations obtained from the simulations withR**_***0***_*** = 1000*,E**_***0***_*** = 776* and varying initialA**_***0***_** numbers.** The dashed arrows denote the fluxes due to enzymes **A** (11↦3) and **E** (21↦5). An increase in **A**_0_ increases the turnover rate, observed by comparing the ratio of the flux and the time interval length. The reactions that correspond to the numbers are given in Figure 4.

In terms of net-flux, these simulations deliver the more dominant flux pathway in the network at steady state as depicted in the left-most graph in Figure 7, where the dashed arrows denote the fluxes due to enzymes **A** (11↦3) and **E** (21↦5). As depicted in Figure 3, this observation is in agreement with the results of [10] with the exception that the reaction flux path **RT**⇒**RD** is not included in our analysis in contrast to the results in [10].

It is important to note that the reaction fluxes in [10] are defined between network species as depicted in Figure 3. As we discuss below, our notion of flux conserves the information of reaction fluxes between species. However, in our stochastic setting, we talk about flux if there is a flow of resources between two reactions. Because of this, the presence of a flux in the pathway from **RT** to **RD** could be explained only with the presence of the reaction **RTE**→**RT**+**E** (21) to the reaction **RT**→**RD** (18). Although this flux can be read, for example, in Sim. 3 in Figure 6 with a weight of ×7 in the flux configuration F[2,2.5] (21↦×718), it is much smaller in weight in comparison to average flux in F[2,2.5] and N[2,2.5].

**Figure 8:**
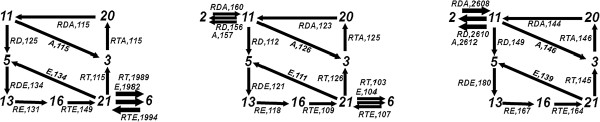
**The graphs displaying the flux configurations obtained from the simulations withR**_***0***_*** = 1000*,E**_***0***_*** = 776* and varying initialA**_***0***_** numbers.** Left: **A**_0_ = 1, F[2,2.5](0.1); Mid: **A**_0_ = 10, F[1.7,1.8](0.1); Right: **A**_0_ = 100, F[0.3,0.4](0.1). The reactions that correspond to the numbers are given in Figure 4.

Another important aspect of this analysis is that the reaction **RTE**→**RT** + **E** (21) and its reverse reaction **RT** + **E**→**RTE** (6) deliver a strong cyclic flux in F[2,2.5], as depicted in Figure 8, which cancels itself in N[2,2.5]. However, this flux has an impact on the network, which we discuss in the next section.

When we carry the analysis above to the simulations where **A**_0_ is increased to 10 and 100, we get the simulation plots depicted in the middle and right-hand-side of Figure 5. We observe the same flux pathway patterns for these simulations with respect to the net-flux configurations, as depicted in the middle and right-hand graphs in Figure 7. However, for the case of flux configurations, as depicted in Figure 8, these regimes introduce other cycles: the **A**_0_ = 100 regime has a strong cyclic flux given with **RDA**→**RD** + **A** (11) and its reverse reaction **RD** + **A**→**RDA** (2), which cancel each other in the net-flux configuration. The **A**_0_ = 10 regime has besides this flux also the cyclic flux given with **RTE**→**RT** + **E** (21) and its reverse reaction **RT** + **E**→**RTE** (6), similar to the **A**_0_ = 1 regime. These fluxes are not considered in the ODE analysis as depicted in Figure 3.

As reported in [10], **E** and **A** play distinct and separable roles in cycling control: the activity (**RT** / **R**_0_) is mainly delivered by **E**_0_ and the turnover rate is a function of **A**_0_. The increase in **A**_0_ does not only decrease the **RT** activity, but also increases the turnover rate, which can be seen by comparing the ratio of the fluxes and the length of the time intervals. This symmetric situation in fluxes with respect to **A** quantities, which we discuss below, can be an explanation for these observations.

It is important to observe that the graphs depicted in Figure 7 are generated from the simulations with the network. Along these lines, Goryachev and Pokhilko argue that a subset of the fluxes of the original network is significant in the actual biological system, while the remaining fluxes have negligible values, and substantiate this prediction by comparing the reduced network with the original model. In this respect, the graphs given in Figure 7 depict a reduced network and agree with the predictions of [10] with the exception of **RT**⇒**RD** as discussed above. This reduced network is obtained by including only those reactions that are included in these graphs. The reduced network suggested by our flux analysis is in agreement with the reduced network given in [10]. Moreover, the graphs in Figure 7 and the reduced network is generated automatically by stochastic flux analysis, and their delivery does not require a further analysis of the network or the modeled biological system. Because our notion of flux is based on flow of resources between reactions, it provides a quantitative means to observe the causality within the system dynamics. Moreover, the stochastic nature of the approach makes it plausible also for the simulations where the quantity of certain species can be arbitrarily small.

### The role of cyclic reverse reactions in network behavior

**Figure 9:**
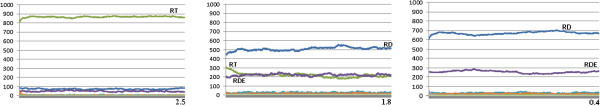
**Example simulation plots of the reduced network obtained from the model in Figure**3**by means of the net-flux analysis, depicted in Figure**7**.** Here, the initial numbers of the species are set to steady state values of the simulations in Figure 5. From left to right, there are 1, 10 and 100 **A** in the network. The reactions are **RE**→**RTE**, **RTE**→**RT** + **E**, **RT** + **A**→**RTA**, **RTA**→**RDA**, **RDA**→**RD** + **A**, **RD** + **E**→**RDE** and **RDE**→**RE**.

The net flux configurations demonstrate the dominant tendencies of the Rho GTP-binding proteins network that are in agreement with the results in [10] with respect to the ODE analysis of the same network. However, when we compare the time series plots of the simulations of this network with those of the reduced network that we obtain from the net-flux analysis (consisting of the reactions 3, 5, 11, 13, 16, 20 and 21), we do not get a satisfactory agreement between them. This can be observed by comparing the time series plots of the simulations in Figure 5 with the beginning of the simulations in Figure 9, where the reduced network diverges from the steady state values of the complete network, with which it has been initiated. In fact, [10] provides a comparison of the reduced and complete networks in terms of histograms that display their activity, where this shift in behavior can be observed as well.

When we consider the simulations with respect to flux configurations instead of net-flux configurations, it is possible to get a different description of the network’s behavior as it is exemplified in Figure 6. This is because there can be strong fluxes in a flux configuration, which do not appear in a net-flux configuration since they cancel each other. However, these fluxes can play an important role for tuning the behavior of the network during simulation. This is because these fluxes have a greater weight in comparison to the others, and they thus shift the simulation resources, thereby causing a shift in the time series of the simulation.

Consider the flux configuration F[2,2.5](0.1) for the Sim. 3 in Table 1, which is depicted on the left-hand-side of Figure 8. We employ a cut-off value of 0.1 with respect to the analysis in Table 2 on the same 10 simulations as in Table 1. In contrast to the net flux configuration on the left-hand-side of Figure 7, the flux configuration exposes a cyclic flux, given with 21↦**RT**6, 21↦**E**6 and 6↦**RTE**21. In order to understand the role of this cyclic flux, we ran simulations with a network, which extends the reduced network (consisting of the reactions 3, 5, 11, 13, 16, 20 and 21) with the reaction 6 (**RT** + **E**→**RTE**). This is because the reaction 6 is included in the flux configuration analysis, although it is excluded by the net-flux analysis.

**Table 2:** **Simulation results with respect to the average flux of**F[2,2.5]** and the number of fluxes given with number of edges in**F[2,2.5](x)** with respect to various cut-off values**

**Sim.**	**Avg.**	**Cut-off value**x→|F[2,2.5](x)|								
		0.02	0.03	0.04	0.05	0.06	0.07	0.08	0.09	0.1
1	213	22	21	21	17	14	12	12	12	12
2	212	23	19	16	13	13	12	12	12	12
3	195	24	24	20	19	19	14	13	12	12
4	222	22	20	18	14	14	12	12	12	12
5	202	23	21	17	12	12	12	12	12	12
6	254	21	17	15	12	12	12	12	12	12
7	224	23	20	18	15	15	14	12	12	12
8	208	23	19	16	13	12	12	12	12	12
9	199	27	22	19	17	12	12	12	12	12
10	261	18	17	16	13	12	12	12	12	12

The initial number of species are R_0_= 1000, E_0_= 776, A_0_= 1.

**Figure 10:**
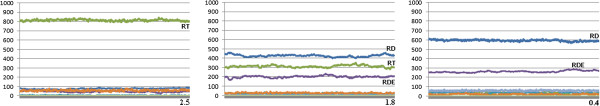
**Example simulation plots of the reduced networks obtained from the model in Figure**3**by means of the flux analysis, depicted in Figure**7**.** The initial numbers of the species are set to steady state values of the simulations in Figure 5. From left to right, there are 1, 10 and 100 **A** in the network. In addition to the reactions of the network in Figure 9, the network for the 1**A** case includes the reaction **RT** + **E**→**RTE**, the network for the 100**A** case includes the reaction **RD** + **A**→**RDA**, and the 10**A** case includes both reactions.

Example simulation plots of the reduced network with reaction 6 are depicted in Figure 10. This network and the complete network have identical flux configuration structures when sufficiently high cut-off values that are in accordance with Table 2 are used. That is, the flux configurations of the original network and this network provide flux behaviors with identical structures with the cut-off values that provide a convergence in the number of fluxes in Table 2. As it can be seen by comparing the example simulation plots of this network in Figure 10 with those in Figure 5 and Figure 9, this network is closer to the complete network also in time-series behavior.

We made the same observations also for the cases, where **A**_0_ = 10 and **A**_0_ = 100. However, as it can be seen in Figure 8, the simulations at the **A**_0_ = 100 regime expose in addition only the cyclic flux, given with 11↦**RD**2, 11↦**A**2 and 2↦**RDA**11, whereas the **A**_0_ = 10 regime expose this cycle as well as the one given with 21↦**RT**6, 21↦**E**6 and 6↦**RTE**21. Although these fluxes are not captured in the reaction flux analysis of [10], the observations made on time-series plots such as those depicted in Figure 5, Figure 9 and Figure 10 suggest that these cycles play an important role in fine tuning the behavior of the network.

By approximating the sample fluxes as normal distributions, we measured the sample mean and variance of the stochastic fluxes on sets of 25 simulations for the three cases of initial **A** levels, given in the Additional file 1. For any given error factor, we computed the probability that the sample mean and variance differ from the true mean and variance by at most the error factor. With this analysis with a sample size of 25, we get estimates of the true mean and variance, accuracy of which can be improved by increasing the sample size. An implication of this analysis is that the variance seems to increase as *A*_0_ gets larger, which may indicate a kind of instability in the system.

### Comparing the notions of flux

We are using the term flux to refer to the flow of resources, that is, the flow of network species, between reactions. However, in deterministic ODE models of chemical reaction networks, it refers to the rates of the reactions themselves. This latter version of flux is often called “reaction flux,” and we follow this convention to distinguish it from our concept. In the following, we show how reaction flux can be defined within our framework.

Consider a reaction network with species *s*_1_,*s*_2_,…,*s*_*n*_ and reactions numbered 1,…,*m*. We write the reactions in the canonical form

(2) $$l_{1,j}s_{1}+\cdots +l_{n,j}s_{n}\to r_{1,j}s_{1}+\cdots +r_{n,j}s_{n},$$

meaning reaction *j* consumes *l*_*i*,*j*_ instances of species *s*_*i*_ and produces *r*_*i*,*j*_ instances of species *s*_*i*_, for *i* = 1,…,*n* and *j* = 1,…,*m*. The effect of all the reactions is summarized by the *n*×*m* stoichiometric matrix *A*. Letting *a*_*i*,*j*_ denote the element in row *i* and column *j* of *A*,

$$a_{i,j}=r_{i,j}-l_{i,j},$$

the effect of reaction *j* on species *i*.

To compute the net effect of a sequence of reactions on the state of the network, let the state at time *t* be given by the column vector *x* = (*x*_1_,…,*x*_*n*_)^′^, where ^′^ denotes the transpose of a matrix or vector, and *x*_*i*_ is the population size of species *i* at time *t*, for *i* = 1,…,*n*. Suppose that in the time interval [*t*,*t*+*Δt*], there are *u*_*j*_ instances of reaction *j* in this sequence, for *j* = 1,…,*m*, and each step in the sequence is feasible, i.e., all the *x*_*i*_ are large enough to avoid any population size becoming negative. Then, letting *u*=(*u*_1_,…,*u*_*n*_)^′^, the state at time *t* + *Δt* will be

(3) x+Au.

*A* is not a complete description of the network because it does not specify the reaction probabilities. As described in the definitions, each reaction *j* has an associated rate constant *ρ*_*j*_. To obtain the total rate of reaction *j*, *ρ*_*j*_ is multiplied by the number of possible ways of selecting the reactants. If the state is *x*, the total stochastic rate of reaction *j* is

(4) vj=∏i=1nxili,j×ρj.

Mass-action kinetics assumes that for small time intervals *Δt*, the average number of occurrences of reaction *j* is approximately *v*_*j*_*Δt*. Therefore by (3), the average state at time *t* + *Δt* is approximately

(5) x+AvΔt.

The classical approach to modeling reaction networks is to approximate them by their average behavior. Essentially, the stochastic rates (4) are treated as deterministic rates called *reaction fluxes*, and the transition equations summarized by (3) are approximated by ODE’s. In general, states in ODE models are concentrations of agents rather than numbers of agents, and the equations must be altered by coefficients that depend on the volume in which the reactions take place. For simplicity, we assume a unit volume. Then, taking *Δt*→0 in (5), the dynamics of the network can be approximated by the system of ODE’s

$$\frac{\text{dx}}{\text{dt}}=\mathrm{Av.}$$

To define reaction flux within our framework, consider a simulation trajectory, and let *n*_*j*_[ *t*,*t*^′^] be the number of vertices of the form (*j*,*τ*) in its simulation configuration such that *t*≤*τ*≤*t*^′^. That is, *n*_*j*_[ *t*,*t*^′^] is the number of times reaction *j* is applied in the time interval [*t*,*t*^′^], and

(6) $$\frac{n_{j}[\;t,t^{\prime }]}{t^{\prime }-t}$$

is the rate at which reaction *j* has occurred over the interval [ *t*,*t*^′^]. Although (6) is a random variable, in the deterministic ODE approximation of the network, as *t*^′^-*t*→0, it approaches *v*_*j*_, the reaction flux of *j* at time *t*.

The term “reaction flux,” as used by Goryachev and Pokhilko, is actually a variation on the definition given by (4). For any reaction *j*, let *j*^′^ be the reverse reaction. That is, if *j* is given by the reaction (2), then *j*^′^ is

$$r_{1,j}s_{1}+\cdots +r_{n,j}s_{n}\to l_{1,j}s_{1}+\cdots +l_{n,j}s_{n}.$$

In some cases, the rate of *j*^′^ is 0, and it is usually omitted from the list of reactions. Goryachev and Pokhilko take the reaction flux of *j* to be $v_{j}-v_{j}^{\prime }$. For the cases, where *v*_*j*_ and $v_{j}^{\prime }$ have relatively close values, this expression results in a negligible flux, which explains the omission of the cyclic fluxes in [10].

### Other examples

In this section, we apply our flux analysis to networks of models from biology and ecology as illustrative case studies.

#### An oscillator

**Figure 11:**
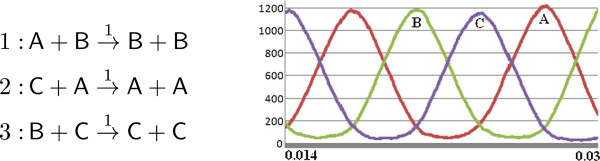
**The reactions of a simple oscillator [**[12]**] and a time series plot of a simulation initiated with A**_***0***_ ***= 900*, B**_***0***_ ***= 500* and C**_***0***_ ***= 100*.**

Below we consider a network that models an oscillator [12]. In the simulations with this network, the amounts of different species increase and decrease at periodic time intervals. Because of this we compare the fluxes of a simulation at these different time intervals. The reactions of this network are given in Figure 11 together with a time series plot of this simulation for the interval 0.014 to 0.03. Here, the initial quantities are A_0_ = 900, B_0_ = 500 and C_0_ = 100, and the species A, B and C increase and decrease periodically during the simulation. In our flux analysis, we consider the individual time intervals, where each of A, B and C increase and decrease. For the increase, we consider the following time intervals:

$$\;\begin{array}{lll} \text{increases} & \left({\text{A}}_{t},{\text{B}}_{t},{\text{C}}_{t})\to ({\text{A}}_{t^{\prime }},{\text{B}}_{t^{\prime }},{\text{C}}_{t^{\prime }}\right) & \left[\;t,t^{\prime }\right]\\ \text{A}\;: & \left(53,789,656)\to (1224,165,110\right) & \left[\;0.0224,0.0274\right]\\ \text{B}\;: & \left(640,53,807)\to (183,1180,137\right) & \left[\;0.0160,0.0208\right]\\ \text{C}\;: & \left(983,466,51)\to (204,136,1160\right) & \left[\;0.0187,0.0242\right] \end{array}$$

**Figure 12:**
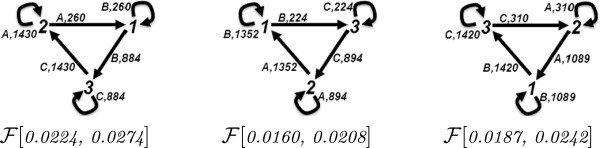
**Flux configurations of the simulation with the oscillator network depicted in Figure**11**for different time intervals, where the species A, B and C increase.**

**Figure 13:**
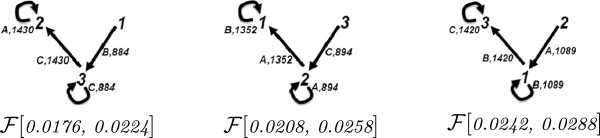
**Flux configurations obtained from those in Figure**12**by applying a cut-off value of 0.35.**

The flux configurations for these time intervals are depicted in Figure 12. In all three flux configurations, the reactions that produce the increasing species receive a stronger flux in comparison to others that feed these reactions with resources. As depicted in Figure 13, a cut-off value of 0.35 removes the smaller fluxes, while preserving the similarities in the structures of the graphs Figure 12. A cut-off value of 1.2 results in a similar situation where, for example, for the case of species A, only the fluxes resulting from the resource flow from reaction 3 to reaction 2 remain.

When we consider the time intervals where each species decreases, we obtain, for example, the following time intervals:

$$\;\begin{array}{lll} \text{decreases} & \left({\text{A}}_{t},{\text{B}}_{t},{\text{C}}_{t})\to ({\text{A}}_{t^{\prime }},{\text{B}}_{t^{\prime }},{\text{C}}_{t^{\prime }}\right) & \left[t,t^{\prime }\right]\\ \text{A}\;: & \left(1185,136,179)\to (53,789,656\right) & \left[0.0176,0.0224\right]\\ \text{B}\;: & \left(183,1180,137)\to (836,58,606\right) & \left[0.0208,0.0258\right]\\ \text{C}\;: & \left(204,136,1160)\to (835,635,30\right) & \left[0.0242,0.0288\right] \end{array}$$

**Figure 14:**
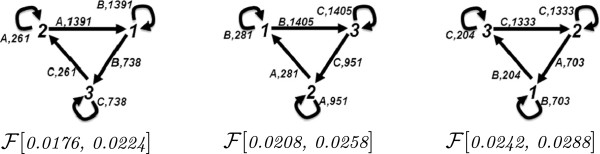
**Flux configurations of the simulation with the oscillator network depicted in Figure**11**for different time intervals, where the species A, B and C decrease.**

The flux configurations for these time intervals, depicted in Figure 14, deliver similar observations to those made with respect to flux configuration in Figure 12. In all three flux configurations for the considered non-steady-state time intervals, the reactions that cause a decrease in species receive a stronger flux in comparison to others.

#### Oyster reef ecosystem

The oyster reef model network [13,14] describes the flow of matter between components in terms of first order reactions, that is, there is only one species on the left-hand-side of the reactions, and also on the right-hand-side of the reactions. The reactions are listed in Figure ??. We ran 20 simulations, where we initiated the simulations with the following steady state values: Filter_Feeder_0_ = 2000, Dep_Detritus_0_ = 1000, Microbiota_0_ = 3, Meiofauna_0_ = 24, Dep_Feeder_0_ = 16, Predator = 69. In the simulations, there is a multiplicity of the reactions with smaller propensities. We observed that for the time interval of 0 to 290, all the 20 simulations result in flux configurations with identical structures with the cut-off value 0.2. The reaction flux graph of this network, as described in previous subsection, is depicted on the left-hand-side of Figure ??. A representative flux configuration of these 20 simulations is depicted on the right-hand-side of Figure ??.

**Figure 15:**
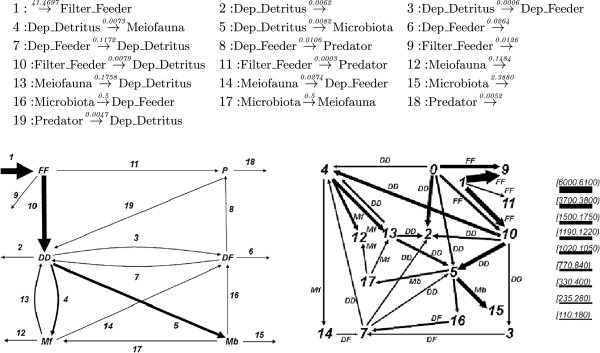
**The reactions of the Oyster Reef network, the reaction flux graph and a representative flux configuration**F[0,290](0.2)** of 20 simulations with this network.** In the reaction flux network on the left, each node represents a species as indicated by the labels. For example, FF is Filter Feeder, Mf is Meiofauna, etc. Each arrow represents a reaction, and its label is the reaction number. The thickness of each arrow is proportional to the reaction count. In the flux configuration graph on the right, each node is a reaction, and each arrow denotes the flux as the flow of the resource in the label. The thickness of each arrow is proportional to the flux strength.

It is important to note that the flux configuration in Figure 15 quantifies the flow of specific species between specific reactions and how these species are consumed and produced by cycling in the network between different reactions. This analysis provides the causality information between reactions in terms of their dependencies, which cannot be revealed by simple reaction counting. In this respect, the causality information does not only quantify the dominant reactions, but also makes the flow of the system resources more explicit by distinguishing their distribution between different reactions, while taking stochasticity into the picture. For example, the flux configuration in Figure 15 clearly shows the flow of DD from producing reactions 7, 10, 13 to consuming reactions 2, 3, 4 and 5, whereas the reaction flux diagram shows only the total activities of the reactions. As discussed in the previous subsection the stochastic flux analysis can be used to construct the reaction flux graph, however constructing flux configurations from reaction graphs is more challenging, in particular for the time intervals that are not in steady state and for the networks that do not consist of only first order reactions.

Based on the flux configuration in Figure 15, we designed a reduced network, which excludes the reactions 6 and 8. From 20 simulations that we performed with this network, two simulations provided flux configurations with identical structures with the cut-off value 0.2 at the time interval 0 to 290 when compared with the flux configurations of the complete network. The disagreement with the eighteen other simulations is because at cut-off value 0.2, sixteen of these simulations prune the flux 10⇒3, and two of them prune also the flux 1⇒11.

#### Phosphorelay

Phosphorelays are signaling networks that are found in various biological systems. We analyze the network given in [15], which models the biochemical phosphorelay mechanism, shuttling the phosphate group from the first to the last layer. The reactions of the network are as follows.

$$\begin{array}{ll} 1:\text{L1}+\text{B}\overset{\text{0.01}}{\to }\text{L1p}+\text{B} & \;\;\;\;\;\;\;2:\text{L1p}+\text{L2}\overset{\text{0.1}}{\to }\text{L1}+\text{L2p}\\ 3:\text{L2p}+\text{L3}\overset{\text{0.1}}{\to }\text{L2}+\text{L3p} & \;\;\;\;\;\;\;4:\text{L3p}+\text{L4}\overset{\text{0.1}}{\to }\text{L3}+\text{L4p}\\ 5:\text{L4p}\overset{\text{1.0}}{\to }\text{L4} \end{array}$$

Reaction 1 describes the phosphorylation of L1 by B. Reactions 2, 3 and 4 describe the transmission of the phosphate to the other levels. Reaction 4 describes the dephosphorylation of L4.

**Figure 16:**
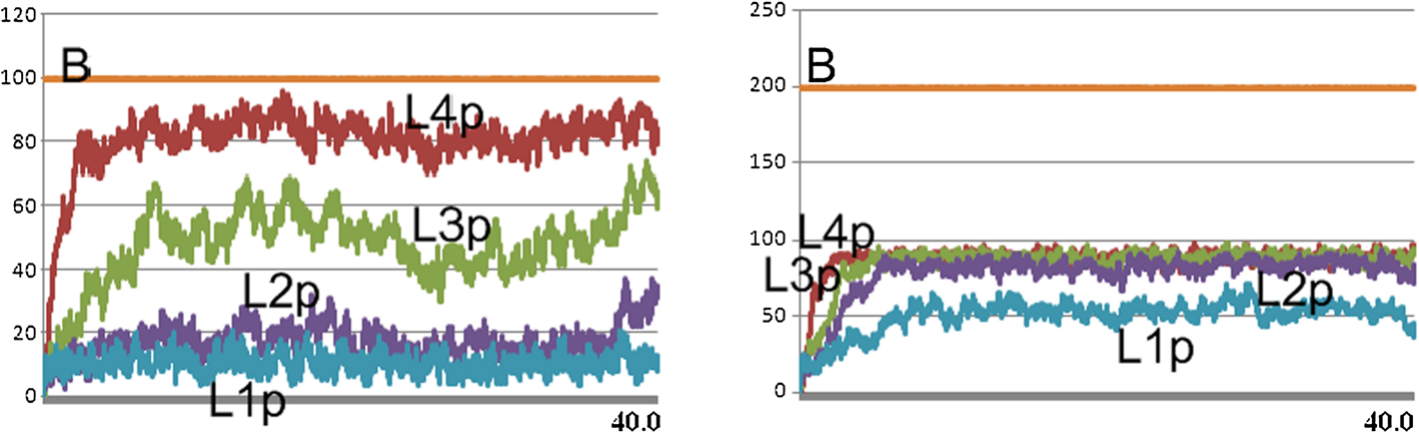
**Time series plots of simulations with the phosphorelay network with 100 L1, L2, L3 and L4 at the initial state.** The simulation on the left is initiated with 100 B, whereas the one on the right is initiated with 200 B.

**Figure 17:**
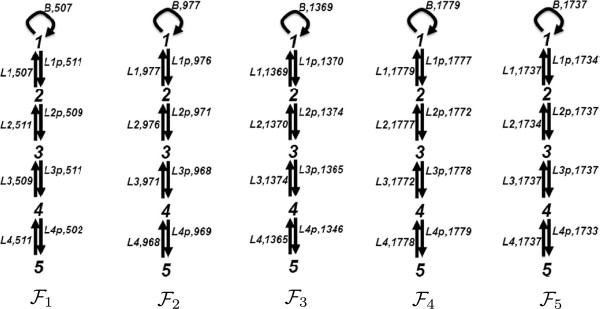
**The flux configurations of the simulations with the phosphorelay network.** From left to right, the simulations are performed with 25, 50, 75, 100 and 200 B at the initial state.

We ran simulations with 100 L1, L2, L3 and L4 at the initial state. We analyzed the steady state fluxes of this network with different input signals, given with the quantity of B in the network. The time series plots of the simulations for 100 and 200 B are depicted in Figure 16. The flux configurations for the time interval 20 to 40 are depicted in Figure 17. These flux configurations, from left to right numbered from 1 to 5, are obtained from simulations with 25, 50, 75, 100 and 200 B. We observe in all five flux configurations that the fluxes are equally distributed at the steady state through out the simulation. We observe that up to 100 B, the turnover rate is directly proportional with the quantity of the B in the network. However, the turnover rate with 200 B approximates the turnover rate of the flux configuration with 100 B.

**Figure 18:**
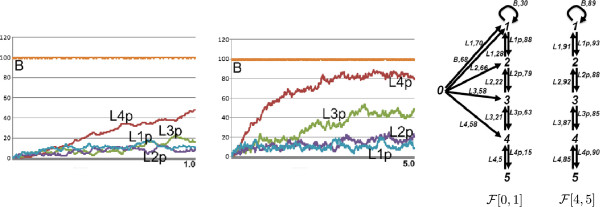
The time series plots of a simulation with the phosphorelay network for the intervals [0,1] and [0,5], and the flux configurations for the time intervals [0,1] and [4,5].

We analyzed the fluxes from the time point 0 to 5, where the network is not in steady state yet, for the 100 B case. The time series plot of a simulation and the flux configurations for the time intervals [ 0,1] and [ 4,5] are depicted in Figure 18. At the beginning of the simulation, the network is biased towards lower-levels, which feed the higher levels. This is because the phosphorylation of the higher-levels requires the phosphorylation of the lower-levels.

### Related work

Flux analysis is well established for the continuous simulations of chemical reaction networks. In this respect, there are many studies dedicated to flux analysis that exploit the differential equation representation of these systems to provide different insights on a variety of aspects from system behavior to model reduction, see, e.g., [7,22-26]. As in [24], these studies also include considerations of deterministic representations of flux analysis for explaining the behavior of stochastic systems. Extensive earlier studies include a series of papers, where Schuster and colleagues give a theory of flux in biochemical networks that is based on linear equation systems, e.g., [27]. In this setting, a flux mode is defined as a steady-state distribution in which the proportions of fluxes are fixed. The fluxes are then computed by using linear algebraic operations to detect all elementary flux modes, which are defined as minimal sets of enzymes that can operate at steady state with all reverse reactions proceeding in the direction prescribed thermodynamics.

Being inspired by studies on non-interleaving semantics of concurrent systems, the current study aims at providing a purely stochastic interpretation of flux in chemical reaction networks. Partial orders reflecting interdependencies and causal relationships in computations have been extensively studied within non-interleaving models of concurrency [28] such as event structures [29]. For sequences of computations in such systems [30] presents an algorithm for extracting partial orders that exhibit event structure semantics. Based on these ideas, preliminary results of the current paper have been presented in [31]. There, we have applied the algorithm of [30] to **SPiM** models of closed systems for flux analysis, where in each reaction a single species can be traced.

The relationship between stochastic models and causality has been studied by various authors. In [32], Danos *et al* draw connections between computational models of biological systems, event structures and their causality interpretation, while considering conflict as a mechanism of inhibition in signalling pathways. In [33], Curti et al apply the ideas presented in [34] to the *π* calculus models of biological systems where the causality information on the modeled system is retrieved by labeling the syntax tree of the process expressions. Probabilistic model checking is another approach, which shares goals with this work. Model checking has been applied to realistic biological examples, e.g., [35], however the state of the art in exhaustive CTMC analysis does not scale well to large systems. Along these lines, in [36], Ballarini *et al* introduce preliminary ideas on an approach for flux analysis by sampling the probabilistic weights of transitions in CTMCs, which however does not scale to larger models due to exponential size of these structures.

An approach, which is closely related to our method is Kazancı and Tollner’s particle tracking method for analyzing ecosystem models [37]. The particle tracking algorithm extends the Gillespie algorithm with a mechanism that labels each species with a unique id, and randomly picks one at each simulation step. In this method dynamic systems, which can be expressed as stock-flow diagrams, can be analyzed. The reactions are thus restricted to single species on the left and on the right-hand-side.

## Conclusions

We have presented a method for flux analysis in stochastic simulations of chemical reaction networks. Our notion of flux provides a precise means for monitoring the flow between reactions, which is different from the flow between species as it is the case in the deterministic setting. Because of this, our approach provides an accurate account of causality within the system dynamics. Because it is applied on stochastic simulations, it can be employed in simulations where species numbers can be arbitrarily small. Moreover, the analysis is not restricted to steady state, but it can be performed on arbitrary time intervals of the simulations as in the case of the oscillator network above, and these intervals can involve arbitrarily big or small number of events. While greater number of events provide more convergent observations, smaller number of events highlight the stochastic nature of the simulations.

The algorithms for generating the data structures of our method apply not just to Gillespie algorithm, but to any discrete event simulator. And not only are they linear in space and time, but the simulation trace can be generated in real time. That is, because the information on resource consumption-production can be recorded in a bounded amount of time during simulation in real time, the method does not introduce any additional complexity to the simulation algorithm. This also suggests our approach as an alternative to model checking of networks with larger CTMCs due to its linear complexity, contrasting with the state-space explosion problem that stochastic model checking faces.

Our steady state analysis of the Rho GTP-binding proteins agree with some of the observations of the analysis in [10]. However, our analysis also introduces new observations: while being in agreement with the notion of flux used in the ODE analysis in [10], the net-flux structures, which counteract the influence of reversible reactions, do not succeed in providing a satisfactory means for identifying the network fluxes that give rise to the time series behavior. In contrast, flux configurations, which also take into account the flow of different species between reactions, permit the observation of all the fluxes, including the cyclic and enzymatic ones, which influence the dynamic behavior of the network. In addition, our analysis displays the effect of different initial conditions that highlight the dominant effect of certain fluxes at different regimes. These different initial conditions give rise to cycles of reverse reactions that shift the simulation resources, thereby adjusting the time-series behavior.

The data structures obtained by the flux analysis algorithm permit the observation of different aspects of the simulations. While the labels of the fluxes as species make it possible to apply label filters for filtering out the fluxes of certain species, the cut-off values make it possible to threshold the fluxes of a chosen relative strength. This also provides the means for obtaining reduced networks from a given network by excluding the reactions that are not included in the flux configurations with a chosen cut-off value. Because increasing the cut-off values results in pruning greater number of fluxes with their reactions, the choice of a cut-off value provides a quantitative means for comparing networks and simulations. The cut-off values employed in comparing the fluxes above can also be seen as a confidence measure, since establishing a similarity between compared simulations for a smaller cut-off value can be perceived more reliable. We have employed a cut-off function that is based on the average fluxes of the system. However, different notions of cut-off can be more appropriate for different systems, which remains a topic of future investigation.

Topics of future research include investigations on the influence of different aspects of reaction networks such as the relative contribution of structure and non-linearity to the dynamical behavior of the system. Although strong non-linearity does not always imply variability in behavior for the stochastic systems, in some cases, and often due to small molecule numbers, stochastic systems can have quite different behaviors from the deterministic ones [38]. In this respect, stochastic analysis can provide novel observations due to the fluxes in comparison with the deterministic approach. Investigations with a more statistical nature can also provide an insight to this discussion as the data analyzed by our algorithms is generated by Monte-Carlo simulations. In this respect, a confidence measure on the results of the analysis as in other Monte-Carlo simulations can provide estimates to reach a desired level of confidence.

## Methods

The algorithm for stochastic flux analysis is applied to a class of dynamical systems that we call interaction systems. The state of an interaction system is a finite set of agents, where each agent has certain attributes that determine the interactions that it is capable of and their likelihood. Well-known examples are discrete stochastic models of chemical kinetics, where each agent has exactly one attribute–the molecular species that it belongs to. More general systems may have agents with additional attributes, such as the presence or absence of methylated sites, attached phosphoryl groups, and other conformational traits. These systems use only finitely many agent attributes, and each attribute has a finite range of values. Therefore the attributes can be encoded by a single attribute with a finite range, and we will restrict our attention to this class of models without loss of generality. Formally, an *interaction system* is a dynamical system whose states and transitions are defined as follows. Let *B* be a fixed finite set (the set of attribute values). A *state* is a pair 〈*A*,*S*〉, where 

• *A* is a finite set of *agents*. Without loss of generality, we can assume *A*⊆{1,…,*n*} for some n∈ℕ.

• *S*:*A*→*B* where, for any *a*∈*A*, *S*(*a*) denotes the value of *a*’s attribute.

We often use  to denote a state. Isomorphism on states is defined in the usual way: two states 〈*A*,*S*〉 and 〈*A*^′^,*S*^′^〉 are isomorphic if there is a 1-1 function *f* mapping *A* onto *A*^′^ such that for every *a*∈*A*, *S*(*a*) = *S*^′^(*f*(*a*)). State transitions are defined by a finite set of *reactions*, numbered 1,…,*m*. Each reaction is a pair $\left(A_{l},A_{r}\right)$ where $A_{l}=\langle A_{l},S_{l}\rangle$ and $A_{r}=\langle A_{r},S_{r}\rangle$ are states, as above. In a network of chemical kinetics, $A_{l}$ and $A_{r}$ are the reactants and products respectively of a reaction.

A *transition* is the application of a reaction to a state, i.e., the occurrence of a reaction. To apply the reaction $\left(A_{l},A_{r}\right)$ to state A=〈A,S〉, a subpopulation of  isomorphic to $A_{l}$ is removed from *A*, and a subpopulation of new agents isomorphic to $A_{r}$ is added. We do not permit the re-use of agents that have been removed; that is, the new agents must be in ℕ-A. Further, in a sequence of states $A_{0},A_{1},A_{2}\ldots \;$ where each $A_{i+1}$ is the result of applying some reaction to $A_{i}$, the new agents must be in $\mathbb{N}-\overset{i}{\underset{j=0}{⋃}}A_{j}$. This is easily implemented by assigning the agents in the initial state $A_{0}$ integers 1,…,*n* for some n∈ℕ, and then using an operator **new** whose successive invocations return the values *n*+1,*n*+2,….

Formally, the reaction $\left(A_{l},A_{r}\right)$ can be applied to the state 〈*A*,*S*〉, resulting in 〈*A*^′^,*S*^′^〉 if: 

• There is an embedding *μ* from $A_{l}$ into . That is, $\mu :A_{l}\overset{}{\underset{1-1}{\to }}A$, and for every *a*∈*A*_*l*_, *S*_*l*_(*a*) = *S*(*μ*(*a*)). (If *μ* does not exist, then the reaction may not be applied.)

• There is an embedding *μ*^′^ from $A_{r}$ into $A^{\prime }$.

• The agents in *μ*^′^(*A*_*r*_) are new in the above sense.

• *A*^′^ = (*A*-*μ*(*A*_*l*_))∪*μ*^′^(*A*_*r*_).

• For all *a*∈*A*∩*A*^′^, *S*(*a*) = *S*^′^(*a*).

A transition is a random event whose probability is determined by the current state of the system. Thus interaction systems are Markov chains. Depending on their implementation, they operate in continuous or discrete time. Models of chemical kinetics implemented by the Gillespie algorithm [39] are continuous time Markov chains. Each reaction has a rate parameter. The time of occurrence of the next reaction and the choice of reaction are random variables determined by the population sizes of the various species and the reaction rates. In contrast, for example, StochSim [40,41] models run in discrete time. At each step, the reactants are chosen randomly, and then a lookup table is used to compute the probabilities of the possible reactions. These probabilities are used to select the next reaction (if any) that will occur.

### **Example5**.

Consider the enzyme-aided reaction

A+B→A+C

which consumes a molecule of type *B* and creates a molecule of type *C*, with the aid of enzyme *A*. In our formalism, 

• $A_{l}=\{1,2\}$, *S*_*l*_(1) = *A*, and *S*_*l*_(2) = *B*.

• $A_{r}=\{3,4\}$, *S*_*l*_(3) = *A*, and *S*_*l*_(4) = *C*.

If this reaction is modeled in continuous time with a rate parameter *ρ*, then for any state , the total reaction rate is *ρm*_1_*m*_2_, where *m*_1_ and *m*_2_ are the population sizes of species *A* and *B* respectively in .

This example also illustrates the main difference between interaction systems and Petri nets. In the former, all agents are distinguishable because each one is identified by a unique integer, and the state of the system is given by the values of *S*(*a*) for each *a*∈*A*. In a Petri net, the agents (tokens) are distinguished only by their species attributes, and the state of the net is given by the markings of the place nodes, i.e., the number of agents in each species. The ability to distinguish all agents enables a precise accounting of the resource usage.

Since interaction systems subsume stochastic Petri nets and numerous other related continuous time Markov chains, and also discrete time Markov chains such as StochSim, our flux analysis applies to all these cases. The first step in the analysis is to build a list of the events that occurred during a simulation, their effect on the state of the system, and when they occurred.

### **Definition6**.

Assume an interaction system starts in some initial state and undergoes *T* transitions at times *τ*_1_<*τ*_2_<⋯<*τ*_*T*_. For *t*=0,…,*T* we put $A_{t}$ for the state after transition *t*, *t* = 0 indicating the initial state. The *T-step simulation trajectory* generated by this sequence of transitions is the sequence of quadruples (*j*_*t*_,*L*_*t*_,*R*_*t*_,*τ*_*t*_), *t* = 1,…,*T*, where 

• *j*_*t*_∈{1,…,*m*} is the index of the reaction applied at transition *t*. Let the reaction be $\left(A_{l},A_{r}\right)$, *μ* be the embedding from $A_{l}$ to $A_{t-1}$, and *μ*^′^ be the embedding from $A_{r}$ to $A_{t}$, as described above.

• *L*_*t*_ = *μ*(*A*_*l*_) and *R*_*t*_ = *μ*^′^(*A*_*r*_).

• *τ*_*t*_ is the time at which transition *t* occurs.

As discrete event simulators generate files listing the events of the simulation with their time stamps, the definition of simulation trajectory above can be incorporated in such discrete event simulators. This is because during a simulation updating the reaction products with unique identifiers requires constant time. The algorithms for generating the data structures discussed here can thus be applied to any discrete event simulator, and not only are they linear in space and time, but these structures can be generated in real time.

### **Notation7**

For ease of presentation, in the examples of the results section and in the examples below, we write the reactions as in Example 5, and states as sets of species where each species in the set is parameterized with a unique $k\in {\mathbb{N}}^{+}$ that denotes an agent. For example, to denote an agent with the identifier 1 of species A that is present at time 0, we write *A*(1,0,0) or, when it is more convenient, *A*(1). Here, the first 0 in *A*(1,0,0) is the identifier of the reaction that created the agent 1, and the second 0 is the time of creation.

The next data structure that we construct reveals the causality relationships between events in the simulation trajectory. This is done by highlighting in a graph structure the implicit production-consumption relationship between reaction instances of the simulation trajectory.

### **Definition8** (simulation trace).

Given an initial state $A_{0}$ and a *T*-step simulation trajectory , their *simulation trace*$K(A_{0},T)$ is a directed acyclic graph (dag) where each vertex has a label (*i*,*j*,*τ*), where $i\in {\mathbb{N}}^{+}$ is an agent identifier, 0≤*j*≤*m* is the index of the reaction that created *i* (0 if the agent is present in the initial state), and *τ*∈[0,*∞*) is the time of its creation.  is defined by induction on *T*: 

• For *T*=0, $K(A_{0},\emptyset )$ consists of vertices labeled (*i*,0,0) where *i* ranges over all agents in *A*_0_.

• Assume  is a *T*-step simulation trajectory, and $K(A_{0},T)$ has been constructed. Let $T^{\prime }$ be the concatenation of  and (*j*_*T* + 1_,*L*_*T* + 1_,*R*_*T* + 1_,*τ*_*T* + 1_). To construct $K(A_{0},T^{\prime })$, for each agent *i*∈*R*_*T* + 1_, add the new vertex (*i*,*j*_*T* + 1_,*τ*_*T* + 1_) to $K(A_{0},T)$. We then add |*L*_*T* + 1_×*R*_*T* + 1_| directed edges from each vertex in $K(A_{0},T)$ with a label of the form (*k*,*j*_*t*_,*τ*_*t*_), *k*∈*L*_*T*+1_, to the new vertices.

### **Example9**.

Consider the simulation trajectory given in Example 1. We obtain the following simulation trace, depicted in Figure 1.

$$\begin{array}{ll} \{\langle (1,0,0), & (2,1,0.46)\rangle ,\\ \;\langle (1,0,0), & (3,1,0.46)\rangle ,\\ \;\langle (2,1,0.46), & (5,2,1.13)\rangle ,\\ \;\langle (3,1,0.46), & (4,3,0.73)\rangle ,\\ \;\langle (5,2,1.13), & (6,4,1.86)\rangle ,\\ \;\langle (4,3,0.73), & \left(6,4,1.86)\rangle \;\right\} \end{array}$$

A simulation trace contains a complete description of the simulation trajectory that generated it, but it also reveals other information about the system, e.g., an explicit record of all the production/consumption relationships in a simulation. In this regard, a simulation trace contains raw data that can be further processed to analyze the simulations. For example, by further processing a simulation trace we can obtain a data structure, that is, an edge-labeled directed multigraph, which contains the independence and causality information of the transitions with respect to the flow of specific resources between them.

An edge-labeled multigraph is a structure 〈*V*,*E*,*L*〉 where *V* is a set of vertices, *E* is a multiset of directed edges on *V*, and *L*: *E*→*D* is a labeling function for some set *D*. *D* is the set of edge labels, and we write 〈*x*, *y*, *c*〉 to indicate that the label of edge 〈*x*, *y*〉 is *c*, i.e., *L*〈*x*, *y*〉 = *c*. In our case, the edge labels are species of the system.

### **Definition10** (simulation configuration).

Given a trace , its *simulation configuration* is the edge-labeled directed multigraph obtained by applying the projection *p*(*i*,*j*,*τ*)=(*j*,*τ*) to the vertices of  and *q*〈(*i*_1_,*j*_1_,*τ*_1_),(*i*_2_,*j*_2_,*τ*_2_)〉=〈(*j*_1_,*τ*_1_),(*j*_2_,*τ*_2_),*S*(*i*_1_)〉 to the edges of , where the label of edge 〈(*j*_1_,*τ*_1_),(*j*_2_,*τ*_2_),*S*(*i*_1_)〉 is *S*(*i*_1_).

Thus each vertex of a simulation configuration shows the reaction that is applied at a particular time, and each edge is labeled with the species that is produced by the reaction at the tail of the edge and consumed by the reaction at its head.

### **Example11**.

From the simulation trace, given in Example 9 we obtain the following simulation configuration, depicted in Figure 1.

(7) $$\begin{array}{lll} \{\langle (0,0), & (1,0.46), & A\rangle ,\\ \;\langle (1,0.46), & (2,1.13), & P\rangle ,\\ \;\langle (1,0.46), & (3,0.73), & P\rangle ,\\ \;\langle (2,1.13), & (4,1.86), & B\rangle ,\\ \;\langle (3,0.73), & (4,1.86), & C\rangle \;\} \end{array}$$

The simulation configuration provides the causality information on the network with respect to the production-consumption relationships between the reaction instances of the simulation, including only those species that are consumed by a reaction. This causality information is revealed by recording *L*_*t*_ and *R*_*t*_ in the simulation trace and passing it on to the simulation configuration. By compressing the simulation configuration, we obtain a structure that we call flux configuration. A flux configuration displays the information on the quantity of species that flow between reactions at chosen time intervals.

### **Definition12** (flux configuration).

Let  be a simulation configuration and $t,t^{\prime }\in {\mathbb{R}}^{+}$ with *t*≤*t*^′^. The *flux configuration* of  between *t* and *t*^′^, denoted by $F[\;t,t^{\prime }]$, is the edge-labeled directed graph where: 

• Its vertices are those reaction indices *j* such that (*j*,*τ*) is a vertex of  for some *τ*∈[*t*,*t*^′^].

• Its edges are those 〈*j*,*j*^′^,*s*,*n*〉 such that there are *n* edges in  of the form 〈(*j*,*τ*),(*j*^′^,*τ*^′^),*s*〉 where *t*≤*τ* and *τ*^′^≤*t*^′^. (That is, the label of edge 〈*j*,*j*^′^〉 is (*s*,*n*).)

The item (*i*.) states that we merge the vertices in  such that all the vertices with reaction *j* that occur between *t* and *t*^′^ are mapped to a single vertice by filtering out their time stamps, that is, *τ*. The item (*ii*.) states that we count the number of edges from each reaction *j* to each reaction *j*^′^ with label *s* between time *t* and *t*^′^. If there are *n* such edges in , we then have an edge 〈*j*,*j*^′^,*s*,*n*〉 in the flux configuration.

### **Example13**.

As in Example 2, consider the simulation configuration depicted in the middle-part of Figure 2. This simulation configuration is obtained from the chemical reaction network in Example 1 and with the initial state {*A*(1),*A*(2),*A*(3),*A*(4)}. The resulting flux configuration, depicted in Figure 2, has the vertices 0, 1, 2, 3 and 4, and its edges are 〈0, 1, *A*, 4〉, 〈1, 2, *P*, 5〉, 〈1, 3, *P*, 3〉, 〈2, 4, *B*, 3〉 and 〈3, 4, *C*, 3〉.

### **Definition14** (net-flux configuration).

Given a flux configuration $F[\;t,t^{\prime }]$, let  be the set of labeled edges 〈*j*,*j*^′^,*m*〉 where

$$m=\text{max}\{\;n\;|\;\exists s\;\langle j,j^{\prime },s,n\rangle \text{is an edge of}F[\;t,t^{\prime }]\;\}.$$

Then, the net-flux configuration $N[\;t,t^{\prime }]$ is the edge-labeled directed graph with the same vertices as $F[\;t,t^{\prime }]$ and with edges 〈*j*,*j*^′^,*k*〉 such that

$$\begin{array}{lll} & \left[\langle j,j^{\prime },k\rangle \in X\wedge ∄n\langle j^{\prime },j,n\rangle \in X\right]\;\vee \; & \;\\ & \left[\exists m,n\langle j,j^{\prime },m\rangle \in X\wedge \langle j^{\prime },j,n\rangle \in X\wedge m>n\wedge k=m-n\right]\; & \; \end{array}$$

## Endnotes

^a^ The article [1] is a letter where Lotka states that he had already published a set of equations, citing a book published in 1925. Volterra had published the equations earlier in an Italian journal, but the article [2] in Nature is the one usually cited.

## Competing interests

The authors declare that they have no competing interests.

## Authors’ contributions

OK designed the algorithms and the data structures for stochastic flux analysis, implemented the models in SPiM and the analysis software in OCaml, and used the software to analyze the stochastic flux of the examples. JL drafted the comparison of stochastic flux with reaction flux. The authors collaborated on the writing. Both authors read and approved the final manuscript.

## Supplementary Material

Additional file 1 **The stochastic mean and sample variance of the stochastic fluxes on sets of 25 simulations for the three different cases of initialAnumbers of the Rho GTP-binding proteins model network.** Click here for file
