# Supplementary material for: Stochastic flux analysis of chemical reaction networks
Source: BMC Syst Biol. 2013 Dec 7;7:133. doi: 10.1186/1752-0509-7-133 (PMC3878955; doi:10.1186/1752-0509-7-133)
Supplement: Additional file 1 — The stochastic mean and sample variance of the stochastic fluxes on sets of 25 simulations for the three different cases of initialAnumbers of the Rho GTP-binding proteins model network. [file 1752-0509-7-133-S1.pdf]

Stochastic Flux Analysis of Chemical Reaction Networks Additional file

Ozan Kahramanoğulları*,[1] James F. Lynch[2]

[1]The Microsoft Research - University of Trento Centre for Computational and Systems Biology, Trento, Italy
[2]Department of Computer Science, Clarkson University, Potsdam, NY, USA

Email: Ozan Kahramanoğulları*- ozan@cosbi.eu;

*Corresponding author

By relying on the Central Limit Theorem and assuming that flux is approximated by a normal distribution with unknown mean μ and unknown variance σ^2, we use repeated simulations to estimate the mean and variance on the data given in Table 1, Table 2, and Table 3. We obtained the mean and variance values in Table 4, which result in the following observations:

1. The probability that the sample mean is within 0.1 standard deviation of the true mean is 0.383. The probability that the sample variance is within 0.1 standard deviation of the true variance is 0.5. Since these probabilities are independent, the probability that both the sample mean and variance are within 0.1 standard deviation of their true values is $0.383 \times 0.5 = 0.1915$.

2. The probability that the sample mean is within 0.2 standard deviation of the true mean is 0.6826. The probability that the sample variance is within 0.2 standard deviation of the true variance is 0.84. Since these probabilities are independent, the probability that both the sample mean and variance are within 0.2 standard deviation of their true values is 0.5734.

3. The probability that the sample mean is within 0.5 standard deviation of the true mean is 0.9876. The probability that the sample variance is within 0.5 standard deviation of the true variance is 0.9997. Since these probabilities are independent, the probability that both the sample mean and variance are within 0.5 standard deviation of their true values is 0.9874.

Sims.	1	2	3	4	5	6	7	8	9	10	11	12	13	14	15	16	17	18	19	20	21	22	23	24	25
$11 \xrightarrow{\mathsf{A}} 3$	132	115	132	128	136	117	122	133	133	127	137	134	116	134	131	122	120	124	135	124	140	126	124	131	117
$21 \xrightarrow{\mathsf{E}} 5$	143	134	154	143	141	146	119	142	142	140	160	158	124	135	133	144	122	138	138	133	142	133	125	147	133
$21 \xrightarrow{\mathsf{E}} 6$	1955	1982	1907	2131	2107	1960	2016	2003	1988	1974	1988	1955	2028	2009	2056	2037	2057	2037	2061	1997	1978	1989	1994	2035	1993
$11 \xrightarrow{\mathsf{RD}} 5$	125	125	137	129	129	134	110	130	127	127	145	138	107	127	126	133	116	127	130	123	136	130	122	132	125
$20 \xrightarrow{\mathsf{RDA}} 11$	131	115	132	127	135	118	121	132	133	127	137	133	116	134	131	123	120	124	135	124	139	127	124	132	118
$5 \xrightarrow{\mathsf{RDE}} 13$	154	134	147	134	122	139	136	143	145	131	139	148	126	142	120	133	125	153	133	136	131	130	127	152	132
$13 \xrightarrow{\mathsf{RE}} 16$	150	131	144	129	117	138	133	141	153	136	136	147	121	135	121	133	123	150	130	133	123	133	126	152	132
$21 \xrightarrow{\mathsf{RT}} 3$	132	115	132	129	136	117	122	133	133	127	137	135	116	133	131	121	120	124	135	124	140	126	124	133	117
$21 \xrightarrow{\mathsf{RT}} 6$	1958	1989	1911	2135	2112	1961	2016	2009	1991	1978	1992	1961	2035	2011	2060	2034	2065	2041	2065	2002	1984	1990	1995	2040	1998
$3 \xrightarrow{\mathsf{RTA}} 20$	132	115	131	128	136	118	122	132	133	126	138	133	116	135	132	122	120	123	135	124	140	126	123	133	117
$6 \xrightarrow{\mathsf{RTE}} 21$	1951	1994	1930	2141	2109	1959	2029	2014	1986	1976	1984	1965	2037	2019	2049	2037	2065	2048	2062	2001	1983	1998	1984	2025	2002
$16 \xrightarrow{\mathsf{RTE}} 21$	160	149	157	136	134	146	143	145	153	148	153	164	131	143	132	140	133	157	143	146	132	147	135	164	137

Table 1: Flux configuration of 25 simulations with the Rho GTP-binding proteins network with 1 A at the initial state and a cut-off value of 0.1.

Sims.	1	2	3	4	5	6	7	8	9	10	11	12	13	14	15	16	17	18	19	20	21	22	23	24	25
$11 \xrightarrow{A} 2$	157	166	150	174	154	159	184	152	185	153	155	151	169	162	140	156	192	159	178	156	151	157	179	170	151
$11 \xrightarrow{A} 3$	126	123	119	105	126	123	110	124	119	114	122	125	123	120	111	115	106	118	104	121	125	120	125	109	118
$21 \xrightarrow{E} 5$	111	124	96	130	120	120	114	110	123	110	115	106	116	113	125	129	127	122	129	116	114	115	100	118	112
$21 \xrightarrow{E} 6$	104	103	132	119	112	127	120	133	98	133	122	115	120	111	113	123	119	109	117	147	123	125	122	112	118
$11 \xrightarrow{RD} 2$	156	162	149	170	152	158	182	150	182	147	155	150	167	162	138	155	191	156	177	154	150	156	180	167	147
$11 \xrightarrow{RD} 5$	112	124	99	127	120	124	115	111	125	111	117	106	119	116	122	128	131	125	127	118	117	121	103	123	113
$2 \xrightarrow{RDA} 11$	160	162	153	171	153	156	186	147	182	152	155	150	170	165	141	156	195	162	173	155	154	151	177	167	153
$20 \xrightarrow{RDA} 11$	123	123	116	107	125	125	107	128	120	113	121	128	123	120	109	116	104	116	104	120	124	121	124	110	117
$5 \xrightarrow{RDE} 13$	121	123	105	136	108	120	107	124	125	108	123	144	106	131	104	110	104	118	122	130	108	115	116	122	127
$13 \xrightarrow{RE} 16$	118	119	101	126	123	119	112	126	122	106	118	144	109	127	91	108	96	111	119	119	114	109	119	117	132
$21 \xrightarrow{RT} 3$	126	123	119	105	126	122	110	124	117	114	121	124	123	120	111	115	106	118	104	121	125	119	124	107	118
$21 \xrightarrow{RT} 6$	103	105	137	121	115	129	123	136	99	135	126	116	121	115	119	123	121	113	122	151	126	126	123	116	120
$3 \xrightarrow{RTA} 20$	125	125	119	106	125	122	110	124	121	114	120	126	122	120	111	116	106	119	104	121	126	120	122	110	118
$6 \xrightarrow{RTE} 21$	107	109	139	126	117	129	124	135	101	127	124	117	117	106	119	123	123	112	123	149	122	126	123	119	119
$16 \xrightarrow{RTE} 21$	109	117	115	133	125	127	113	120	124	114	126	143	106	110	90	114	92	115	130	125	109	114	128	120	129

Table 2: Flux configuration of 25 simulations with the Rho GTP-binding proteins network with 10 A at the initial state and a cut-off value of 0.1.

Sims.	1	2	3	4	5	6	7	8	9	10	11	12	13	14	15	16	17	18	19	20	21	22	23	24	25
$11 \stackrel{A}{\to} 2$	2612	2606	2614	2633	2616	2636	2673	2681	2623	2743	2696	2658	2582	2657	2708	2590	2749	2728	2618	2668	2614	2625	2608	2642	2619
$11 \stackrel{A}{\to} 3$	146	175	164	139	134	151	130	158	144	169	142	164	150	141	153	137	146	142	145	165	136	137	138	154	133
$21 \stackrel{E}{\to} 5$	139	120	142	162	144	151	141	152	156	158	138	151	154	166	168	165	145	140	147	135	147	161	131	130	143
$11 \stackrel{RD}{\to} 2$	2610	2600	2611	2630	2617	2630	2671	2678	2618	2738	2695	2654	2580	2657	2709	2591	2745	2725	2615	2667	2609	2623	2600	2640	2617
$11 \stackrel{RD}{\to} 5$	149	129	155	171	156	168	150	161	166	167	151	166	165	171	178	170	157	156	156	146	159	177	137	140	157
$2 \stackrel{RDA}{\to} 11$	2608	2603	2626	2653	2619	2639	2673	2691	2626	2744	2704	2659	2588	2667	2724	2589	2756	2718	2624	2659	2621	2622	2590	2642	2611
$20 \stackrel{RDA}{\to} 11$	144	179	164	135	135	157	126	154	142	167	140	165	149	138	153	133	145	144	139	165	135	140	139	155	131
$5 \stackrel{RDE}{\to} 13$	180	168	141	153	153	142	155	150	142	157	133	154	145	139	134	138	158	143	162	166	145	149	157	142	138
$13 \stackrel{RE}{\to} 16$	167	165	139	135	158	145	146	155	141	163	143	140	142	140	144	137	154	135	152	153	142	137	157	143	139
$21 \stackrel{RT}{\to} 3$	145	175	160	136	132	150	130	158	143	169	142	163	148	140	153	136	146	140	144	164	136	136	137	152	130
$3 \stackrel{RTA}{\to} 20$	146	175	162	137	134	153	129	156	143	169	140	162	151	139	152	136	146	142	145	165	136	137	138	154	133
$16 \stackrel{RTE}{\to} 21$	164	163	152	135	148	152	140	155	151	168	141	142	146	137	149	147	154	136	149	163	146	128	158	139	140

Table 3: Flux configuration of 25 simulations with the Rho GTP-binding proteins network with 100 A at the initial state and a cut-off value of 0.1.

		$11 \xrightarrow{\text{A}} 2$	$11 \xrightarrow{\text{A}} 3$	$21 \xrightarrow{\text{E}} 5$	$21 \xrightarrow{\text{E}} 6$	$11 \xrightarrow{\text{RD}} 2$	$11 \xrightarrow{\text{RD}} 5$	$2 \xrightarrow{\text{RDA}} 11$	$20 \xrightarrow{\text{RDA}} 11$
$1A$	μ	NA	127.6	138.76	2009.48	NA	127.6	NA	127.52
	σ^2	NA	50.	100.9824	2313.9296	NA	65.04	NA	45.9296
	D	NA	0.3918	0.7277	1.1515	NA	0.5097	NA	0.3601
	c_v	NA	18.0453	13.8083	41.7742	NA	15.8219	NA	18.8162
$10A$	μ	162.4	118.04	116.6	119.08	160.52	118.16	161.84	117.76
	σ^2	166.08	45.9584	72.	109.5136	170.4896	62.3744	164.0544	51.6224
	D	1.0226	0.3893	0.6174	0.9196	1.0621	0.5278	1.0136	0.4383
	c_v	12.6016	17.4119	13.7414	11.3790	12.2936	14.9612	12.6354	16.3899
$100A$	μ	2647.96	147.72	147.44	NA	2645.2	158.32	2650.24	146.96
	σ^2	2113.6384	144.3616	142.0864	NA	2125.68	143.0176	2280.5824	171.7184
	D	0.7982	0.9772	0.9636	NA	0.8035	0.9033	0.8605	1.1684
	c_v	57.5964	12.2945	12.3691	NA	57.3732	13.2385	55.4960	11.2147

		$5 \xrightarrow{\text{RDE}} 13$	$13 \xrightarrow{\text{RE}} 16$	$21 \xrightarrow{\text{RT}} 3$	$21 \xrightarrow{\text{RT}} 6$	$3 \xrightarrow{\text{RTA}} 20$	$6 \xrightarrow{\text{RTE}} 21$	$16 \xrightarrow{\text{RTE}} 21$	
$1A$	μ	136.48	134.68	127.68	2013.32	127.6	2013.92	145.12	
	σ^2	87.7696	100.7776	51.3376	2334.7776	51.12	2251.1136	98.9856	
	D	0.6430	0.7482	0.4020	1.1596	0.4006	1.1177	0.6820	
	c_v	14.5679	13.4159	17.8198	41.6667	17.8465	42.4466	14.5861	
$10A$	μ	118.28	116.2	117.68	121.64	118.08	121.44	117.92	
	σ^2	111.0016	122.24	45.6576	118.9504	42.8736	103.6064	136.9536	
	D	0.9384	1.0519	0.3879	0.9778	0.3630	0.8531	1.1614	
	c_v	11.2265	10.5099	17.4159	11.1530	18.0335	11.9307	10.0762	
$100A$	μ	149.76	146.88	146.6	NA	147.2	NA	148.12	
	σ^2	126.8224	88.8256	148.4	NA	144.8	NA	98.8256	
	D	0.8468	0.6047	1.0122	NA	0.9836	NA	0.6671	
	c_v	13.2983	15.5845	12.0341	NA	12.2327	NA	14.8997	

Table 4: Sample mean (μ) and variance (σ) of the stochastic fluxes with a cut-off value of 0.1 on a sample of 25 simulations, together with their index of dispersion ($D = \sigma^2/\mu$) and coefficient of variation ($c_v = \mu/\sigma$).
